# Cross-Reactive Antibodies Binding to the Influenza Virus Subtype H11 Hemagglutinin

**DOI:** 10.3390/pathogens8040199

**Published:** 2019-10-21

**Authors:** Shirin Strohmeier, Fatima Amanat, Florian Krammer

**Affiliations:** 1 Department of Microbiology, Icahn School of Medicine at Mount Sinai, New York, NY 10029, USA; shirin.strohmeier@mssm.edu (S.S.); fatima.amanat@icahn.mssm.edu (F.A.); 2 Graduate School of Biomedical Sciences, Icahn School of Medicine at Mount Sinai, New York, NY 10029, USA

**Keywords:** avian influenza, monoclonal antibodies, H11, H11N1, H11N2, H11N3, H11N4, H11N5, H11N6, H11N7, H11N8, H11N9, Hav3

## Abstract

H11 subtype influenza viruses were isolated from a wide range of bird species and one strain also was isolated from swine. In an effort to generate reagents for a chimeric H11/1 hemagglutinin-based universal influenza virus vaccine candidate, we produced 28 monoclonal antibodies that recognize the H11 HA subtype. Here we characterized these antibodies in terms of binding breadth and functionality. We found that the antibodies bind broadly to North American and Eurasian lineage isolates and also show broad neutralizing activity, suggesting that immunogenic epitopes on the H11 head domain are not under strong pressure from immunity in the natural reservoir. Furthermore, we found that the antibodies were highly hemagglutination inhibition active against the homologous chimeric H11/1N1 virus, but approximately 50% lost this activity when tested against a virus expressing the same the full length H11 HA of which the head domain is present on cH11/1 HA. Furthermore, while strong neutralizing activity was found to a genetically distant North American lineage H11 isolate, little hemagglutination inhibition activity was detected. This suggests that small structural changes between wild type H11 and cH11/1 as well as between Eurasian and North American lineage H11 HAs can strongly influence the functionality of the isolated monoclonal antibodies.

## 1. Introduction

**Figure 1:**
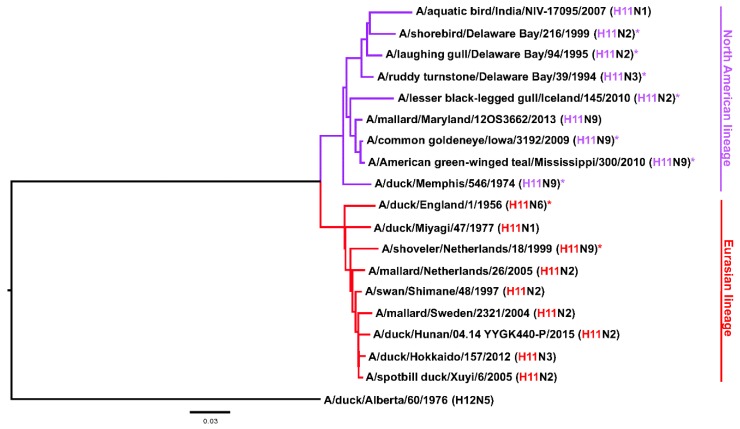
Phylogenetic tree of the H11 HA subtype based on amino acid sequences. H12 was used as an out-group. The scale bar represents a 3% change in amino acid sequence. The North American and Eurasian lineage are indicated. Strains used in this study are marked with *.

The Hav3 subtype was first isolated from a duck in England in 1956 [1,2] and its presence in North America was first shown in 1974 [3]. Later on the nomenclature for hemagglutinin (HA) subtypes was changed and Hav3 became H11 [1]. The H11 subtype circulates in seagulls—like its close relatives H13 and H16—but it has been found quite frequently in ducks and other bird species as well [4,5,6,7,8,9,10]. Information on infection of mammals is limited, however, one strain has been isolated from swine (A/swine/KU/2/2001 H11N6, CY073452.1) and there is serological evidence for exposure of humans to H11 viruses from hunters, wild life professionals [11,12] and from Lebanese poultry workers [13] (but not poultry workers in the US [14]). Exposure in sea otters has also been serologically confirmed [15]. Furthermore, some H11 HAs can recognize both alpha 2,3 and alpha 2,6 sialic acids and at least one isolate has been shown to replicate well and cause weight loss in the mouse model (A/black necked stilt/Chile/2/13, H11N9) [16]. It is also worth noting, that H11 cleavage can occur in a trypsin-independent manner, despite the absence of a polybasic cleavage site [17]. Nevertheless, it can be assumed that this subtype has a low pandemic potential and its ability to grow in mammals seems limited [16,18,19]. Like many avian influenza virus HA subtypes, H11 can be split into two phylogenetic groups, the Eurasian clade and the North American clade (Figure 1). Confusingly, isolates from North America sometimes cluster in the Eurasian clade and *vice versa*, due to the global dispersion of the viruses through migratory birds [5,20]. While H11N2 and H11N9 are the most common isolates [5], H11 has been isolated with all of the *bona fide* NA subtypes (N1–N9) [16,19,21,22,23,24]. Finally, little is known about antibody epitopes of H11. With the purpose of making reagents for stability studies of a chimeric HA-based universal influenza virus vaccine candidate [25,26,27], we generated anti-H11 monoclonal antibodies (mAbs) and characterized them *in vitro*. Here we report our findings and hope that the mAbs are also useful reagents for research and surveillance of H11 subtype influenza viruses.

## 2. Results

### 2.1. Generation of mAbs and Intial Characterization

To generate mAbs, mice were immunized twice with cH11/1N1 virus and then boosted with recombinant cH11/1 HA protein. The cH11/1N1 virus contains the HA head domain of A/shoveler/Netherlands/18/99 (H11N9) and the HA stalk domain of A/California/4/09 (H1N1) and has been developed as a universal influenza virus vaccine candidate [25,26,27]. Three days post boost the spleen was harvested and a hybridoma fusion was performed. After screening for reactivity to cH11/1 recombinant protein and isotyping, 28 IgG producing monoclonal hybridomas were obtained. Eighteen clones were expressing IgG2a, eight IgG2b, one IgG1 and one IgG3 (Table 1). The hybridomas were then grown up in serum free medium and mAbs were purified for further characterization.

**Figure 2:**
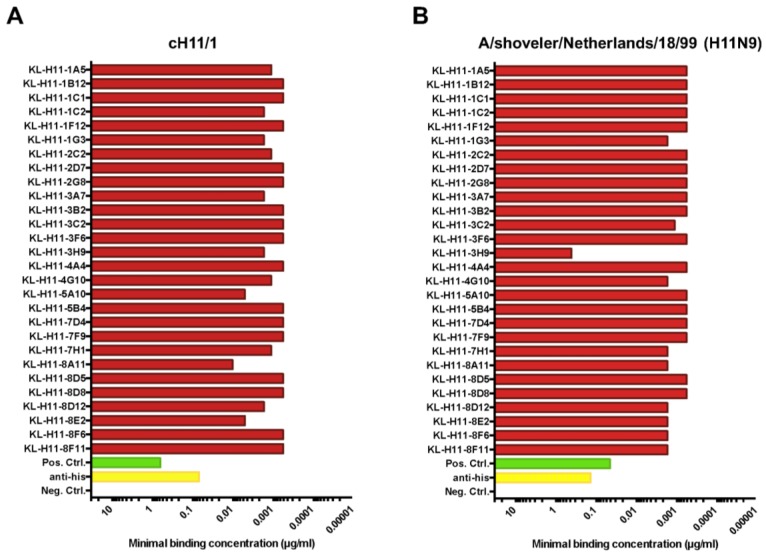
Minimal binding concentration of mAbs to homologous recombinant HA. Minimal binding concentration of anti-H11 mAbs to (**A**) cH11/1 and (**B**) A/shoveler/Netherlands/18/99 H11 HA. Since the cH11/1 carries the head domain of the A/shoveler/Netherlands/18/99 HA, both proteins are homologous to the immunogens used to vaccinate the mice prior to the fusion. MAb CR9114 (pan-HA, stalk binding) and an anti-hexahistidine tag antibody were used as positive control (the recombinant proteins are hexahistidine-tagged). An anti-Lassa virus glycoprotein antibody (KL-AV-IA12) was used as negative control.

For initial characterization, the minimal binding concentration of the 28 mAbs against recombinant cH11/1 HA as well as recombinant H11 HA from A/shoveler/Netherlands/18/99 (which is homologous to the head domain of the cH11/1 HA) was determined in an enzyme-linked immunosorbent assay (ELISA). As shown in Figure 2A, all mAbs exhibited low minimal binding concentrations (meaning good binding) to cH11/1 HA. All except one mAb (KL-H11-3H9) also showed comparable good binding to the recombinant H11 HA of A/shoveler/Netherlands/18/99 (Figure 2B).

### 2.2. Most H11 mAbs Are Cross-Reactive

**Figure 3:**
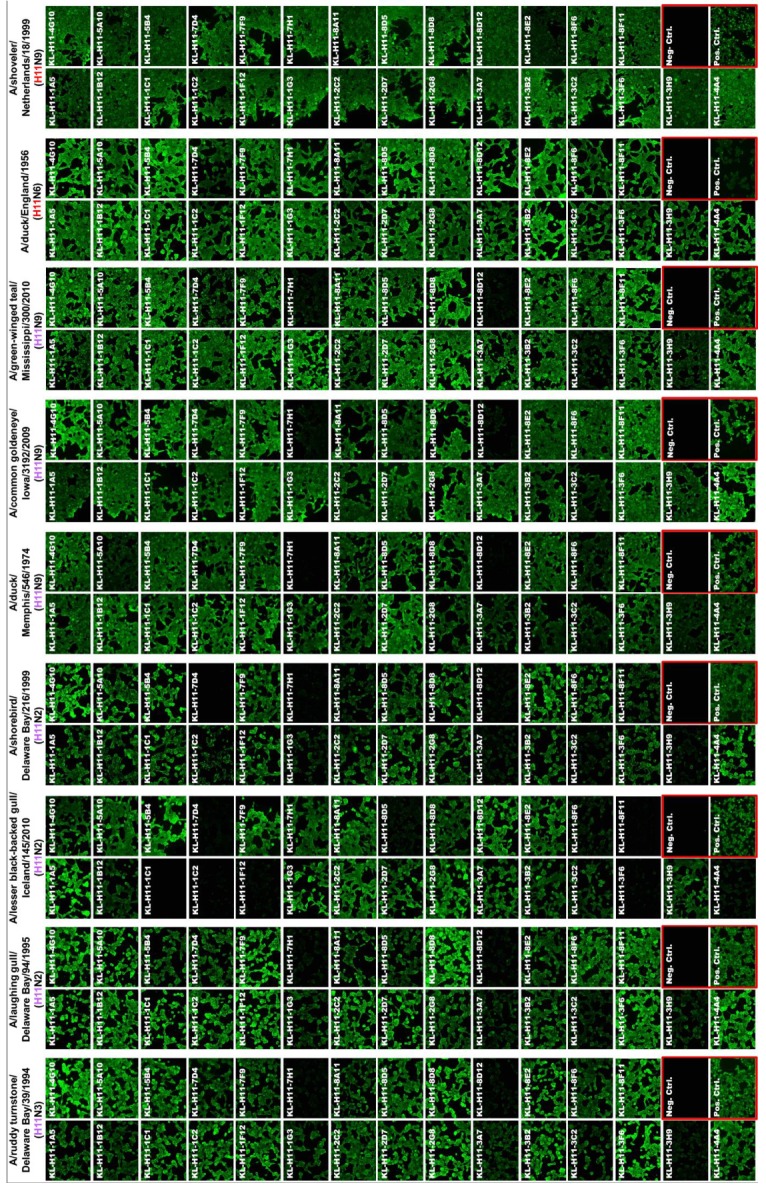
Cross-reactivity of anti-H11 mAbs was tested using immunofluorescence microscopy on virus infected MDCK cells. North American and Eurasian lineage strains are colored according to the color scheme in Figure 1. An anti-nucleoprotein antibody was used as positive control and an anti-Lassa virus glycoprotein mAb (KL-AV-1A12) was used as negative control. The A/shoveler/Netherlands/18/99 (originally H11N9, E lineage) used was a 7:1 A/PR/8/34 reassortant.

Next, we wanted to test to what extend the isolated mAbs would react to different isolates from both the North American (NA) and Eurasian (E) lineage. For this we assembled a panel of H11 subtype viruses including A/ruddy turnstone/Delaware Bay/39/94 (H11N3, NA lineage), A/laughing gull/Delaware Bay/94/95 (H11N2, NA lineage), A/lesser black-backed gull/Iceland/145/10 (H11N2, NA lineage), A/shorebird/Delaware Bay/216/99 (H11N2, NA lineage), A/duck/Memphis/546/1974 (H11N9, NA lineage), A/common goldeneye/Iowa/3192/09 (H11N9, NA lineage), A/green-winged teal/Mississippi/300/10 (H11N9, NA lineage) and A/duck/England/1956 (H11N6, E lineage) in immunofluorescence staining of infected cells. The homologous A/shoveler/Netherlands/18/99 (H11N9, E lineage, used as 7:1 A/PR/8/34 reassortant) strain was included as a control. Like in the ELISA, all mAbs except KL-H11-3H9 bound well to the homologous virus (Figure 3). The same was observed for the second Eurasian-lineage isolate, A/duck/England/1956. Interestingly, more than half of the mAbs cross-reacted with all tested strains and the remaining showed broad binding as well (although they did not bind to all strains).

### 2.3. All H11 mAbs Are HI Active against cH11/1N1 Virus but Not All Are HI Active against HA-Head Homologous Wild Type H11 Virus

**Figure 4:**
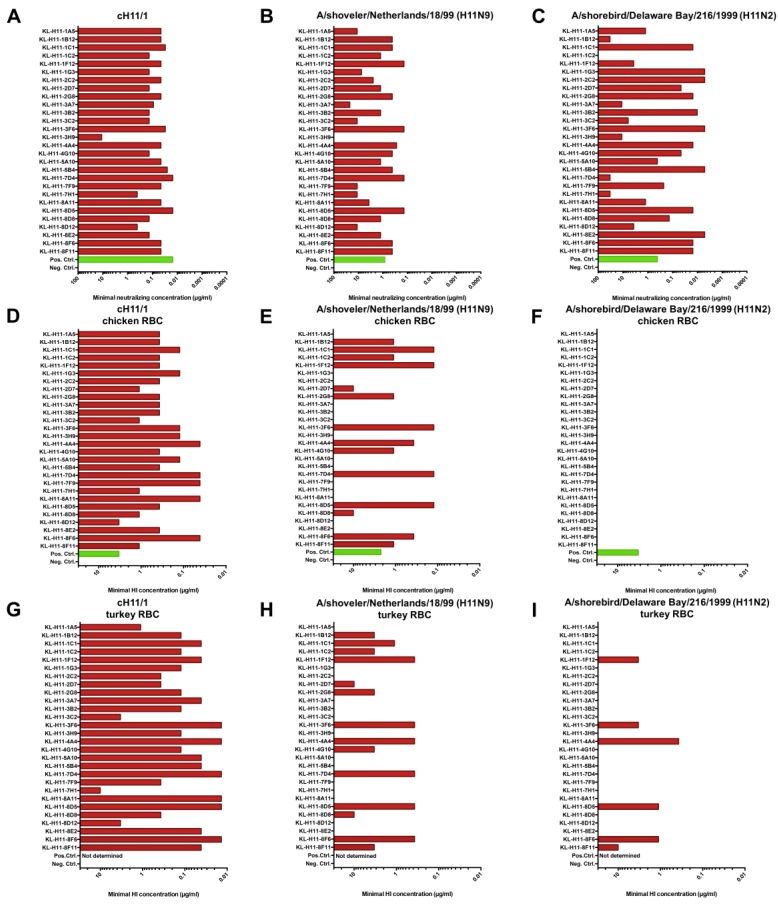
Functionality of isolated mAbs in neutralization and hemagglutination inhibition assays. Neutralization activity of the isolated mAbs against (**A**) cH11/1N1, (**B**) A/shoveler/Netherlands/18/99 and (**C**) A/shorebird/Delaware Bay/216/99. Hemagglutination inhibition activity (measured using chicken red blood cells (RBC)) of the isolated mAbs against (**D**) cH11/1N1, (**E**) A/shoveler/Netherlands/18/99 and (**F**) A/shorebird/Delaware Bay/216/99. (**G**–**I**) shows the same assay with the three respective viruses but performed with turkey red blood cells. Serum from mice vaccinated with cH11/1N1 was used as positive control and an anti-Lassa virus glycoprotein antibody (KL-AV-1A12) was used as negative control. The A/shoveler/Netherlands/18/99 (originally H11N9, E lineage) used was a 7:1 A/PR/8/34 reassortant.

Having characterized binding, we also wanted to assess activity and first performed neutralization assays using the cH11/N1 virus and a reassortant virus carrying the H11 HA of A/shoveler/Netherlands/18/99. This comparison was performed since we have found in earlier studies that the HA head domain of cHAs can have a slightly different conformation than it has on wild type HA owing to the combination with a heterosubtypic stalk domain [28]. Neutralization activity was uniformly strong against the cH11/1N1 virus (Figure 4A) except for KL-H11-3H9 which had somewhat lower potency. Interestingly, the majority of mAbs, while maintaining neutralizing activity, had much lower potency against the wild type H11 HA expressing virus (Figure 4B). Next, we performed a hemagglutination inhibition (HI) assay against the cH11/1N1 virus and the re-assortant virus. All tested mAbs had strong HI activity to the cH11/1N1 virus (Figure 4D,G). Interestingly, only approximately 50% of mAbs retained HI activity against the re-assortant virus carrying the H11 HA of A/shoveler/Netherlands/18/99 (Figure 4E,H), independently of the type of red blood cells (chicken or turkey) used in the assay. Of note, for several of the mAbs that retained activity, the activity was retained at full potency.

### 2.4. Several H11 mAbs Show HI and Neutralizing Activity against An Eurasian Lineage Virus but Only Show Neutralizing Activity against A North American Lineage Isolate

Since many of the mAbs showed neutralizing and HI activity against the homologous Eurasian lineage A/shoveler/Netherlands/18/99 strain, we wanted to assess if this activity would span to a North American lineage strain. For this we tested neutralizing activity and HI activity against A/shorebird/Delaware Bay/216/99. Neutralization activity mostly followed the pattern seen for binding to this isolate in IF assay on infected cells, with some exceptions (Figure 4C). In general, good neutralization activity was observed for a majority of mAbs. Interestingly, none of the mAbs showed any HI activity against the A/shorebird/Delaware Bay/216/99 strain when chicken red blood cells were used (Figure 4F). Some of the mAbs became HI positive against the same strain when the assay was performed with turkey red blood cells (Figure 4I).

### 2.5. H11 mAbs Bind to Linear Epitopes

**Figure 5:**
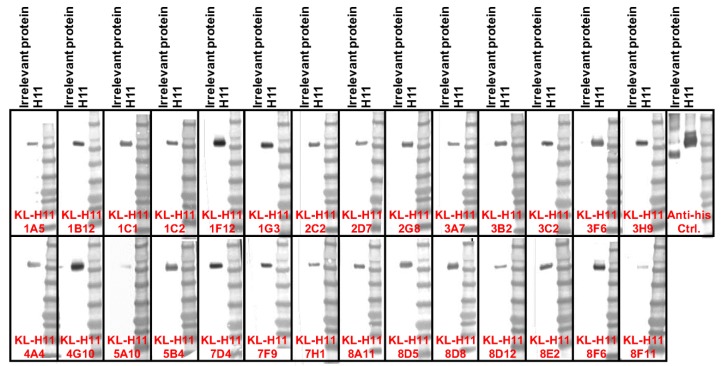
Western blot analysis of the isolated anti-H11 mAbs. Recombinant H11 protein from A/shoveler/Netherlands/18/99 and an irrelevant Junin virus glycoprotein control were run on an SDS-PAGE under denaturing and reducing conditions and a Western blot was performed. An anti-hexahistidine antibody present on both recombinant proteins was used as positive control. All tested mAbs bound to H11 under these conditions.

Finally, we also wanted to learn more about the character of the epitope. To determine if the mAb epitopes are linear/microconformational or tertiary/quarternary we performed a Western blot under denaturing, reducing conditions (Figure 5). Interestingly, all mAbs bound to recombinant H11 HA in this assay, suggesting that they in fact all do target linear or microconformational epitopes.

## 3. Discussion

The initial motivation behind generating the described mAbs was to produce reagents that can be used for characterization and release and identity testing of cH11/1N1 universal influenza virus vaccine candidates and several of the described mAbs were actually used for this purpose [25,26,27]. However, since there is not much known about the antigenicity of H11 HA, based on a paucity of H11 reagents, we decided to characterize the 28 mAbs that we obtained. Interestingly, the antibodies are broadly reactive spanning both the North American and Eurasian lineage with half of the mAbs being pan-H11 binders. Several additional mAbs showed broad binding between the two lineages, even though they did not cover all tested strains. This is despite the fact that they are HI active which indicates that they likely bind to epitopes on the variable head domain of the HA. We hypothesize that this is due to low antigenic drift in the animal reservoir as compared to humans. During their short life, avian hosts might not get infected repeatedly with the same subtype/strain and therefore there is no need for the virus to escape immunity by drifting. While the HA gene certainly genetically changes when replicating in avian hosts, the changes might be less driven by antigenic pressure and more by stochastic effects leading to the preservation of antigenic site sequences. This phenomenon has also been observed with H7 HAs which have a quite conserved antigenic site A despite being split into a North American and Eurasian lineage [29,30]. Similar observations have also been made for H4 HA [31].

The second interesting observation that we made is that the same antibody, e.g., KL-H11-3B2, can have neutralizing activity against cH11/1N1, A/shoveler/Netherlands/18/99 (which is the donor for the head domain of cH11/1 HA) and A/shorebird/Delaware Bay/216/99 but then shows HI activity only against cH11/1N1. In other cases, mAbs neutralize all three viruses but only have HI activity against cH11/1N1 and A/shoveler/Netherlands/18/99 but no HI activity against A/shorebird/Delaware Bay/216/99 (e.g., KL-H11-1C1). The difference between cH11/1N1 and A/shoveler/Netherlands/18/99 (which have both exactly the same HA head domain) might be explained by slight conformational changes between H11 and cH11/1 which are caused by forcing an H11 head and an H1 stalk together. In turn, this could change the angle of approach and steric hindrance that an antibody exerts and therefore might change its ability to block interactions between the receptor binding site and terminal sialic acids on host proteins. In an earlier study we have observed these differences for cH5/1 constructs which had a slightly different structure than both H1 and H5 wild type HAs [28]. The difference between binding to wild type A/shoveler/Netherlands/18/99 and A/shorebird/Delaware Bay/216/99 might be explained by a similar phenomenon, although here it is more likely that the overall conformation is the same but e.g., subtle changes like additional glycans or bulky amino acids cause these differences.

Several of the described mAbs were already useful for vaccine development but we hope that they can also serve in other roles. They could potentially be useful in surveillance and diagnostic kits, might serve as controls in serology and could also serve as controls in HI and virus neutralization assays, ELISAs and Western blots for research with H11 subtype viruses. Further mapping of their epitopes could also lead to a better understanding of H11 immunogenicity.

In summary, we isolated 28 antibodies that broadly bind to the head domain of both North American and Eurasian lineage H11NX viruses. We hope that these mAbs will be useful tools and reagents for future research and surveillance efforts.

## 4. Materials and Methods

### 4.1. Cells and Viruses

Sf9 (*Spodoptera frugiperda*) insect cells were grown in *Trichoplusia ni* medium—Fred Hink (TNM-FH, Gemini Bioproducts) supplemented with 1% penicillin/streptomycin antibiotics mix (100 U/mL of penicillin, 100 µg/mL streptomycin, Gibco, Waltham, MA, USA), 1% Pluronic F-68 (Sigma-Aldrich, St. Louis, MO, USA) and 10% fetal bovine serum (FBS, Gibco). For passaging the baculoviruses in Sf9 cells 3% TNM-FH insect medium (1% penicillin/streptomycin, 1% Pluronic F-68, 3% FBS) was used. BTI-TN-5B1-4 (Trichoplusia ni, High Five) cells were passaged in serum-free SFM4 insect cell medium (HyClone) containing 1% penicillin/streptomycin. Madin-Darby Canine Kidney (MDCK) cells (ATCC #CCL-34) used for various assays were grown in Dulbecco’s Modified Eagle’s Medium (complete DMEM, Gibco) supplemented with 1% penicillin/streptomycin, 10% FBS and 1% hydroxyethylpiperazine ethane sulfonic acid (HEPES, Gibco). SP2/0-Ag14 myeloma cells used for hybridoma fusion were grown and maintained in complete DMEM supplemented with 1% L-glutamine (Gibco). The viruses A/duck/Memphis/546/74 (H11N9;# NR-21661), A/common goldeneye/Iowa/3192/09 (H11N9;# NR-31134), A/duck/England/56 (H11N6; # NR-21660), A/laughing gull/Delaware Bay/94/95 (H11N2;# NR-45183), A/shorebird/Delaware Bay/216/99 (H11N2;# NR-45185), A/American green-winged teal/Mississippi/300/10 (H11N9;# NR-31137), A/lesser black-legged gull/Iceland/145/10 (H11N2;# NR-44393) and A/ruddy turnstone/Delaware Bay/39/94 (H11N3,# NR-45186) were obtained from the Biodefense and Emerging Infections Research Resources Repository (BEI Resources). Two versions of the cH11/1N1 (head domain of A/shoveler/Netherlands/18/99 H11N9 and stalk domain of A/California/4/09 H1N1) [27] virus were used. One was rescued in the A/PR/8/34 backbone (used for mouse immunizations), the second one was rescued in the temperature sensitive cold-adapted A/Leningrad/134/17/57 backbone [32]. The viruses were grown in 10-day-old embryonated chicken eggs (Charles River Laboratories) and the titers determined by performing standard plaque assays [33]. Briefly, 1 × 10^6^ MDCK cells/well were seeded in a sterile 6-well cell culture plate. On the following day, the cells were washed with phosphate buffered saline (PBS) and incubated with the respective virus dilutions for 1 h at 37 °C. The virus was aspirated and the cells overlaid with agar consisting of minimal essential medium (2xMEM), 2% oxoid agar, 1% diethylaminoethyl cellulose (DEAE) and N-tosyl-L-phenylalanine chloromethyl ketone (TPCK) treated trypsin. The plates were incubated at 37 °C for two days and the cells afterwards fixed with 3.7% paraformaldehyde (PFA) in PBS. The plaques were visualized by immunostaining. The recombinant virus A/shoveler/Netherlands/18/99 (H11N9) was rescued with the HA from the original strain and the remaining seven segments from A/PR/8/34 (PR8) as a 7:1 reassortant virus.

### 4.2. Recombinant Proteins

The recombinant H11 (A/shoveler/Netherlands/18/99 H11N9) and cH11/1 (head domain of A/shoveler/Netherlands/18/99 H11N9 and stalk domain of A/California/4/09 H1N1) [27] glycoproteins were generated by using the baculovirus expression system as described previously [34]. Briefly, the HA ectodomains were cloned into a baculovirus shuttle vector, containing a C-terminal T4 trimerization domain and a hexahistidine purification tag. The baculoviruses were amplified in Sf9 cells and then used to infect High Five cells for expression as described in detail before [35] and were stored at −80 °C for further usage.

### 4.3. Enzyme-Linked Immunosorbent Assay

Ninety-six well flat bottom plates (Immulon 4 HBX plates, ThermoFisher Scientific, Waltham, MA, USA) were coated with 50 µL/well of 2 µg/mL recombinant protein in 1x KPL coating buffer (SeraCare, Milford, MA, USA) at 4 °C overnight. The coating solution was removed and the plate blocked with 100 µL/well of 3% milk dissolved in PBS with 0.1% Tween 20 (TPBS) for 1 h at room temperature (RT). The blocking solution was removed and primary antibody dilutions were prepared in 1% milk/TPBS starting at a concentration of 30 µg/mL followed by 1:3 serial dilutions. MAb CR9114 [36] was used as a positive control and an irrelevant anti-Lassa virus glycoprotein antibody (KL-AV-1A12) as negative control [37]. The antibody was incubated on the plate for 2 h at RT, followed by three washes with 100 µL/well TPBS. The plate was incubated with 100 µL/well of anti-mouse secondary antibody (anti-mouse IgG H&L antibody peroxidase conjugated, Rockland, Limerick, PA, USA) diluted 1:3000 in 1% milk/TBPS for 1 h at RT. The plate was washed three times with 100 µL/well of TBPS before adding 100 µL/well of SigmaFast o-phenylenediamine dihydrochloride (OPD) developing solution (Sigma Aldrich). The reaction was stopped after 10 min incubation at RT with 50 µL/well of 3M hydrochloric acid (HCl). The plate was read with a Synergy H1 hybrid multimode microplate reader (BioTek, Vinooski, VT, USA) at an optical density of 490 nm. The data were analyzed by using GraphPad Prism 7 software.

### 4.4. Generation of Monoclonal H11-Antibodies

A female 6–8-week-old BALB/c mouse (The Jackson Laboratory) was first immunized intraperitoneally with cH11/1N1 virus, containing the HA head domain of A/shoveler/Netherlands/18/99 (H11N9) and the HA stalk domain of A/California/4/09 (H1N1), followed three weeks later by a second intranasal immunization with the same virus [27]. The mouse was then boosted four weeks later intraperitoneal with recombinant H11 protein (A/shoveler/Netherlands/18/99) adjuvanted with 10 µg of poly (I:C) (Invivogen). Three days after the final boost, the mouse was euthanized and the spleen removed. The spleen was washed with PBS and then flushed with serum-free DMEM (1% penicillin/streptomycin) using a 10 mL syringe with a 20-gauge needle to obtain the splenocytes. The splenocytes were fused with SP2/0-Ag14 myeloma cells in a ratio of 5:1 using polyethylene glycol (PEG; Sigma-Aldrich). The cells were grown on semi-solid selection and cloning medium with hypoxanthine-aminopterin-thymidine (HAT; Molecular Devices) for 10 days. The resulting colonies were picked and transferred to a 96-well cell culture plate. The protocol used for this procedure has been described in detail [38,39]. The hybridoma supernatant was used to screen for H11-specific antibodies via ELISA and reactive clones were isotyped using the Pierce rapid antibody isoytping kit (Life Technologies, Carlsbad, CA, USA). Only IgG heavy-chain isotype expressing clones were selected. The selected hybridoma clones were first expanded in Clonacell-HY Medium E and then switched to Hybridoma SFM media (Gibco) supplemented with 1% penicillin/streptomycin. The antibodies were purified by affinity chromatography using protein G sepharose columns following an earlier described protocol [40].

### 4.5. Immunofluorescence Assay

MDCK cells were seeded at a density of 50,000 cells/well in a sterile 96-well cell culture plate by using complete DMEM media and were then infected the next day with a multiplicity of infection (MOI) of 1 overnight for 16 h. For this the cell culture medium was switched to serum free minimal essential medium (MEM, Gibco) containing 1 µg/mL TPCK-treated trypsin. The cells were fixed with 100 µL/well of 3.7% paraformaldehyde (PFA) for 1 h at RT. The plate was blocked with 3% milk/PBS for 1 h at RT. The antibodies were diluted to a concentration of 30 µg/mL in 1% milk/PBS and 100 µL/well were added for 1 h at RT. An anti-Lassa virus glycoprotein antibody (KL-AV-1A12) [37] was used as a negative control. An anti-influenza A virus nucleoprotein polyclonal antibody (Invitrogen) was used as positive control, after the cells were permeabilized by using 100% methanol as a fixative. The cells were washed twice with 1x PBS and then incubated for 1 h with a goat anti-mouse IgG heavy plus light chain (H + L)–Alexa Fluor 488 antibody (Abcam, Cambridge, UK) diluted 1:1000 in 1% milk/PBS. Afterwards, the plate was washed three times with PBS and kept in PBS during immunofluorescence microscopy using an Olympus IX-70 microscope.

### 4.6. Hemagglutination Inhibition Assay

Hemagglutination inhibition assays were performed as described in detail earlier [30]. Briefly, a hemagglutination assay (HA) was performed in order to determine the hemagglutination units (HAU) of the respective viruses. The antibody dilutions were prepared at a starting concentration of 30 µg/mL, followed by 1:3 serial dilution. Serum from mice which were vaccinated with cH11/1N1 was used as a positive control and an irrelevant anti-Lassa virus glycoprotein mAb (KL-AV-1A12) [37] was used as a negative control. The H11 and cH11/1 viruses were diluted to eight HAU and then incubated with the antibody dilutions for 1 h shaking at RT. Afterwards, 50 µL/well of 0.5% chicken or turkey red blood cells (Lampire Biological Laboratories) were added to the antibody/virus mixture and the plates incubated for 1 h at 4 °C.

### 4.7. Microneutralization Assay

MDCK cells were seeded at a density of 50,000 cells/well in a sterile flat-bottom 96-well cell culture plate. Antibody dilutions were prepared at a starting concentration of 100 µg/mL and then 1:2 serially diluted. The viruses were diluted to 4000 plaque forming units (PFU)/mL in 1x MEM and 60 µL of that dilution was then incubated together with 60 µL of the antibody dilutions for 1 h shaking at RT. The mixture (100 µL) was added to the MDCK cells and incubated for 1 h at 37 °C. The antibody/virus mixture was aspirated and the cells overlaid with the same antibody dilution as used earlier, containing 1 µg/mL of TPCK treated-trypsin. The cells were incubated for two days at 37 °C for A/shoveler/Netherlands/18/99 and A/shorebird/Delaware Bay/216/99 and for three days at 33 °C for the cH11/1N1 which is a cold adapted virus and grows at lower temperature. Incubation times were optimized for each virus. An HA-assay of the supernatants was performed in order to determine the minimal neutralizing concentration.

### 4.8. Western Blot

For Western blot analysis, 10 ng of recombinant A/shoveler/Netherlands/18/99 H11 were prepared in PBS and mixed 1:1 with 2x Laemmli loading buffer (Bio-Rad, Hercules, CA, USA), which was supplemented with 5% of beta-mercaptoethanol. An anti-Junin virus glycoprotein was used as an irrelevant control. The samples were heated at 95 °C for 15 min prior to loading them on a sodium dodecyl sulfate polyacrylamide gel (SDS-PAGE, 5–20% gradient, Bio-Rad). The gel was afterwards blotted on a nitrocellulose membrane. The membrane was blocked with 3% non-fat milk/TPBS for 30 min at RT and then incubated with 30 µg/mL of the respective H11 mAbs for 2 h at RT. An anti-hexahistidine antibody was used as positive control, since both the H11 and Junin virus glycoprotein contain a hexahistidine-purification tag. The membrane was washed three times with TPBS and then incubated with an anti-mouse IgG alkaline phosphatase (AP) antibody (Sigma Aldrich) for 1 h at RT. The membrane was developed by using an AP conjugate substrate kit (Bio-Rad).

## Figures and Tables

**Table 1:** Names and subtypes of the obtained anti-H11 hybridomas.

Name	Subtype
KL-H11-1A5	IgG2a
KL-H11-1B12	IgG2a
KL-H11-1C1	IgG2b
KL-H11-1C2	IgG2a
KL-H11-1F12	IgG2b
KL-H11-1G3	IgG2a
KL-H11-2C2	IgG2a
KL-H11-2D7	IgG2a
KL-H11-2G8	IgG2a
KL-H11-3A7	IgG2b
KL-H11-3B2	IgG2b
KL-H11-3C2	IgG2b
KL-H11-3F6	IgG2b
KL-H11-3H9	IgG2a
KL-H11-4A4	IgG2a
KL-H11-4G10	IgG2a
KL-H11-5A10	IgG2a
KL-H11-5B4	IgG2b
KL-H11-7D4	IgG2a
KL-H11-7F9	IgG2a
KL-H11-7H1	IgG2a
KL-H11-8A11	IgG2a
KL-H11-8D5	IgG2b
KL-H11-8D8	IgG1
KL-H11-8D12	IgG2a
KL-H11-8E2	IgG2a
KL-H11-8F6	IgG3
KL-H11-8F11	IgG2a
